# The Proper Method of Applying Obstetric Forceps

**Published:** 1892-11

**Authors:** 


THE PROPER METHOD OF APPLYING OBSTETRIC FORCEPS.—1. Anesthetize the patient and place her in proper position—buttocks well over the edge of the bed, and each limb supported by an assistant.

2. Ascerting the position of the head, introducing within the vagina two or three fingers, or if necessary the whole hand.

3. Apply the blades of a Hodge type of forceps to the sides of the head, with the concave edge directed toward the occiput. If for any reason this cannot be accomplished, withdraw the instrument and substitute a Simpson (or Elliott) passing the blades to the side of the pelvis. While making traction with this method, watch for anterior rotation of the occiput, and encourage it in some cases by re-applying the blades to better advantage.

4. Make every effort to secure antiseptic condition during the operation. The fingers, hands and forearms of the operator, the external genitalia and vagina of the patient, the instruments and the hands of the assistants, should be clean and aseptic.—*Amer. Jour. Obstetrics*.—(*Medical Brief.*)

